# Alterations in anthropometric, inflammatory and mental health parameters during Ramadan intermittent fasting in a group of healthy people: a prospective cohort study

**DOI:** 10.3389/fnut.2024.1298281

**Published:** 2024-02-01

**Authors:** Samaneh Khosandam Ghashang, Abdulhadi Suwandi, Manuela Buettner, Imad Hamdan, Guntram A. Grassl, Christoph Gutenbrunner, Boya Nugraha

**Affiliations:** ^1^Department of Rehabilitation and Sport Medicine, Hannover Medical School, Hannover, Germany; ^2^Department of Dermatology, Johannes Wesling Medical Centre, Minden, Germany; ^3^Institute of Cell Biochemistry, Hannover Medical School, Hannover, Germany; ^4^Institute of Medical Microbiology and Hospital Epidemiology, Hannover Medical School, Hannover, Germany; ^5^German Center for Infection Research (DZIF), Partner-Site Hannover-Braunschweig, Hannover, Germany; ^6^Institute for Laboratory Animal Science, Hannover Medical School, Hannover, Germany; ^7^Hannover Rehabilitation Services and Science Consulting, Hannover, Germany

**Keywords:** Ramadan intermittent fasting, neurotrophic factors (NTFs), Interleukin-8 (IL-8), TNF-α – tumor necrosis factor alpha, anthropometric, matrix metalloprotein-9 (MMP-9), mental health parameters and quality of life (QoL) parameters

## Abstract

Fasting has been practiced with different time span in different areas of the world and for various reasons. One of the types of fasting regimens is Ramadan intermittent fasting (RIF), which is described as intermittent dry fasting and known as the most commonly practiced form of religious fasting. Different studies have shown its effects on body composition parameters and mental health, fatigue and quality of life (QoL). Elucidating the relationship of RIF on biological parameters would also be of importance to show its mechanism. Therefore, we evaluated several biological mediators related to mental health, such as ß-nerve growth factor (ß-NGF), brain-derived neurotrophic factor (BDNF), glial cell line-derived neurotrophic factor (GDNF), and insulin-like growth factor-1 (IGF-1), interleukin-8 (IL-8), tumor necrosis factor-α (TNF-α), and matrix-metalloproteinase-9 (MMP-9). This study consisted of fasting (FG; *n* = 25) and non-fasting group (NFG; *n* = 25). Four different time points were assessed for FG: one week before (T1), mid (T2), last days (T3), and one week after (T4) RIF. T1 and T3 were the assessment time points for NFG. Biological mediators were determined from serum samples by using Human Magnetic Luminex and enzyme-linked immunosorbent assay. Furthermore, we then performed correlation analyses between biological mediators and our previously published clinical parameters including body composition and mental health parameters at all time points. Significant alterations were shown in FG for ß-NGF (T2vsT3, *p* < 0.05; T2vsT4, *p* < 0.05), GDNF (T1vsT4, *p* < 0.05; T2vsT4, *p* < 0.05), IL-8 (T2vsT3, *p* < 0.05; T3vsT4, *p* < 0.05), TNF-α (T1vsT3, *p* < 0.05; T1vsT4, *p* < 0.001; T2vsT4, *p* < 0.001), and MMP-9 (T1vsT4, *p* < 0.01). There were no statistically significant differences between FG and NFG in all biological mediators at T1 and T3. Correlation analysis showed that MMP-9 levels had negative correlation with body mass index (BMI) at T3. At T3 BDNF levels had negative correlation with Epworth Sleepiness Scale (ESS) as one of measured QoL parameters. ß-NGF, GDNF, TNF-α, and MMP-9 had positive correlation with some of body composition and mental health parameters. Findings demonstrate that RIF altered different biological mediators could give benefit to health. Its benefit is mediated by the alteration of biological mediators.

## Introduction

Intermittent fasting (IF) has become a lifestyle of eating during specific period of time and fasting for the rest of the day. IF has shown many of the health benefits. It is not only reducing free-radical production and weight loss, but also the adaptive cellular responses between and within organs that restores glucose metabolism, increases stress resistance and suppresses inflammation (1). Previous studies showed the positive effect of IF in animal models on chronic disorders such as obesity, diabetes, cardiovascular disease, cancer and neurological disorders (2, 3). Ramadan intermittent fasting (RIF) is one type of IF that performed by about 1.5 billions of Muslims worldwide annually from dawn to sunset for one month. The time periods of RIF vary from 9 to 22 h per day due to geographical differences and seasonal variation (4). Food, water, smoking and sexual activities are forbidden during the fasting daytime hours of Ramadan month. Our previous studies demonstrated that not only RIF can improve anthropometric parameters, but also can improve mental health, fatigue and quality of life (QoL) (5). However, the physiological mechanism behind these beneficial effects need to be elucidated.

Depression is related to irregularities in hypothalamic–pituitary–adrenal (HPA) axis, altered insulin resistance, plasma glucose, pro-inflammatory cytokines, leptin resistance, and disturbance of neurotransmitters, neuropeptides and neurotrophic factors (6, 7). Interestingly, several studies demonstrated that IF has positive effects on the neuroendocrine system and on depression. IF might induce an antidepressant effect on depression, providing potential new treatment options. It included neurotrophic effects and orexin signaling activation that can release endorphin and ketone via the increased of cAMP response element-binding (CREB) phosphorylation (8). Hence, it is of interest to study the effect of IF on biological mediators related to depression, particularly on neurotrophic factors.

Nerve growth factor (NGF) was the first member described among the neurotrophic factors (9). NGF has been known to involve in the regulation of neurotransmitters and neuropeptides synthesis of nerve cells. It is important for growth, maintenance, proliferation and survival of nerve cells (10). Various clinical studies in human and animals showed an association of low NGF levels with depression compared with healthy controls (11). Brain-derived neurotrophic factor (BDNF), another member of neurotrophic factors, was described as being involved in synaptic plasticity (12), depression (8), and increasing survival of dopaminergic neurons of the developing substantia nigra (13). BDNF has been also studied in different neurological-related diseases, such as major depressive disorders, fibromyalgia syndromes and chronic low back pain (14). Glial cell-derived neurotrophic factor (GDNF) is a small protein and member of neurotrophic factors that are responsible for the development, maintenance and survival of different neurons (15). In mice model, induced GDNF-expression showed protective effects to obesity. In addition, several studies showed that GDNF is known to the mechanism underlying depressive disorders (16).

Other biological mediators related to depression and body composition parameters such as insulin growth factor-1 (IGF-1), tumor necrosis factor alpha (TNF-α), interleukin-8 (IL-8), and matrix metalloproteinase-9 (MMP-9) were also determined (17–19). One of the biological mechanisms for depression is related to inflammation. People with major depressive disorder (MDD) and obesity have been shown to have higher inflammatory markers as compared to healthy control (20–22). IGF-1 has been shown to have anti-inflammatory action by inhibiting the expression of proinflammatory cytokines including IFN-γ, IL-1β and TNF-α and enhancing the production of anti-inflammatory cytokines including IL-4 and IL-10 (23). The abnormalities of IGF-1 in MDD patients have been suggested as a marker and predictive role of the neurotrophin for depression and treatment effectiveness (24). Meanwhile, individuals who had increased level of both pro-inflammatory cytokine tumor necrosis factor-alpha (TNF-α) and interleukin 8 (IL-8) have been reported to develop psychiatric disorders such as major depression, bipolar disorder, schizophrenia and sleep disorder (25, 26). Other study showed that depression condition in young patient, levels of serum MMP-9 were increased which might be used as biomarker for bipolar disorder (27). Another study also showed that patients in acute phase of ischemic stroke condition, serum MMP-9 levels were elevated (28). In addition, animal were treated with MMP-9 inhibitor showed improve specific neurobehavioral deficits associated with Alzheimer’s disease (29).

As mentioned before, neurotrophic factors play a role not only related to depressive disorders, but also to body composition parameters. Therefore, this study was an explorative study that aimed at elucidating the changes in neurotrophic factors upon the observation of RIF. Additionally, we aimed to measure the inflammatory cytokine TNF-α, the chemokine IL-8 and MMP-9. Furthermore, in our previous study showed that RIF improved body composition and mental health parameters, fatigue and QoL (5). For the body composition parameters, we measured body weight (BW), body mass index (BMI), skeletal muscle mass (SMM), body fat mass (BFM), fat free mass (FFM), body fat percentage (BFP), body water mass (BWM) and wait and hip ratio (WHR). For mental health parameters, we determined the effects on fatigue [Fatigue Severity Scale (FSS) and visual analogue scale (VAS)], depression (The Hospital Anxiety and Depression Score (HADS) and Beck’s Depression Inventory (BDI)-II) and QoL [Epworth Sleepiness Scale (ESS)] (5). In this study, we aimed to analyze the correlation between measured biological mediators with our previously published clinical parameters including body composition and mental health parameters at all time points.

## Materials and methods

Local Ethic Committee of Hannover Medical School approved this study (Ethics No. 6899) in accordance with the ethical standards laid down in the 1964 Declaration of Helsinki. This study registered at German Registry of Clinical Trial (DRKS-ID: DRKS00008181). Written informed consent was obtained from all subjects and/or their legal guardian(s).

### Participants

This study was a sub analysis from previous report (5). Healthy male participants which mostly students of Hannover Medical School were recruited. We divided them into two groups fasting group (FG) and non-fasting group (NFG). The health status of participants were assessed by experienced doctors at Department of Rehabilitation and Sport Medicine, Hannover Medical School. Study inclusion for FG required a minimum age of 18 years old, intended to fast the whole month of Ramadan, have fasted during Ramadan at least once before, understood German or English language. Study inclusion for NFG required a minimum age of 18 years old and did not fast during the study period.

### Study design

**Figure 1:**
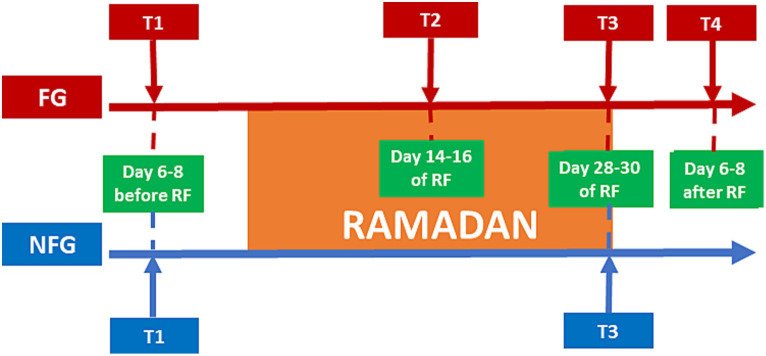
Study design.

In this current study, the fasting period was about 18–19 h per day. In the FG, subjects were assessed at four time points (Figure 1): one week before the beginning of Ramadan/baseline (T1); middle of Ramadan (T2: day 14th or 15th or 16th); the last days of Ramadan (T3: day 28th or 29th or 30th); and one week after the Ramadan fasting finished (T4: day 6th, 7th, 8th from the end of Ramadan).

In the NFG, subjects were assessed only at 2 time points: before Ramadan (T1) and during the last days of Ramadan (T3).

### Serum preparation

Serum was prepared by collecting blood samples from peripheral venous by using Monovette serum tube (Monovette®, Sarstedt, Germany). It was done in the morning time between 08:00 and 10:00 for all time points. Centrifugation was performed after allowing blood clotting at 1500 g for 15 min (Universal 320R, Hettich, Tuttlingen, Germany). These serum samples were stored at −80°C until determination.

ß-NGF, BDNF, GDNF, TNF-α, IL8 and MMP-9 serum level were determined using customized Human Magnetic Luminex Assay (R&D Systems) according to the manufacturer’s instructions. IGF-1 serum levels were determined using Quantikine ELISA Human IGF-1 Immunoassay (R&D Systems Inc.) according to the manufacturer’s instructions.

### Body composition and mental health parameters

As discussed above, body composition and mental health parameters have been published in our previous publication (5). We determined the body composition parameters including body weight (BW), body mass index (BMI), skeletal muscle mass (SMM), body fat mass (BFM), fat free mass (FFM), body fat percentage (BFP), body water mass (BWM) and wait and hip ratio (WHR) by using InBody 230 (Model MW160, InBody Co., Ltd., Seoul, Korea). Mental health parameters, fatigue and QoL were assessed by using self-administered questionnaires. The Hospital Depression and Anxiety Score (HADS) and Beck’s Depression Inventory (BDI)-II were used to assess the intensity of anxiety and depression or only depression, respectively. Fatigue was measured by the visual analogue scale (VAS) and fatigue severity scale (FSS). Day sleepiness as QoL parameter was measured by using the Epworth Sleepiness Scale (ESS). In this study, we only showed mental health parameters that have correlation with measured biological mediators (ESS, HADSA, HADSD, BDI-II).

### Statistics analysis

The aim of this study is to assess the level of ß-NGF, BDNF, GDNF, IGF-1, IL-8, TNF-α, and MMP-9 before (T1), during (T2 and T3) and after (T4) RIF. Kruskal-Wallis or two-way Friedman ranked test were used to analyze the data, after we tested the data distribution using the Kolmogorov–Smirnov test. We performed the *post hoc* tests, and Bonferroni correction were used to adjust the significances. We used Spearman’s correlation tests to determine correlation between clinical parameters and biological mediators. Missing data were replaced by using a mean. Statistical analysis was performed by using SPSS version 22 (IBM, New York, United States).

## Results

### Baseline characteristics of FG and NFG

Table 1 shows the baseline data of FG and NFG regarding anthropometric measurements and mental health parameters of both groups that were measured from our previous publication (5). This data demonstrated that there were no statistically significant differences at baseline between FG and NFG. Table 2 shows the baseline data of FG and NFG regarding biological mediators measured in this study. There were no statistically significant differences at baseline between FG and NFG.

**Table 1:** Baseline data of fasting and non-fasting group (Body composition and mental health parameters).

	FG (*N* = 25)	NFG (*N* = 25)	*p*
	Mean ± SEM	Mean ± SEM	
Age	26.12 ± 0.98	26.20 ± 0.98	0.977
**Ethnicity**			
White/Asian	21/4	16/9	0.196^§^
**Body composition**			
BW (kg)	77.82 ± 2.46	76.16 ± 4.29	0.739
BMI (kg/m^2^)	24.78 ± 0.73	24.56 ± 0.78	0.84
SMM (kg)	34.66 ± 1.02	34.60 ± 1.05	0.972
BFM (kg)	16.65 ± 1.48	17.44 ± 1.68	0.726
FFM (kg)	61.18 ± 1.72	61.10 ± 0.176	0.98
BFP (%)	20.92 ± 1.41	21.54 ± 1.41	0.797
BWM (kg)	44.88 ± 1.25	44.77 ± 1.80	0.96
WHR	0.88 ± 0.04	0.92 ± 0.02	0.293
**Mental health**			
Anxiety (HADSA)	4.92 ± 3.82	4.26 ± 3.38	0.521
Depression (HADSD)	4.36 ± 3.88	3.06 ± 3.47	0.218
Depression (BDI-II)	8.36 ± 8.21	6.48 ± 5.97	0.359
**Fatigue**			
Fatigue (VAS)	3.01 ± 1.83	3.08 ± 1.96	0.893
Fatigue Severity Scale (FSS)	26.92 ± 8.65	26.44 ± 10.38	0.86
Epworth Sleepiness Scale (ESS)	7.96 ± 3.81	7.16 ± 3.73	0.457

All data are presented in Mean ± SEM; body weight (BW), body mass index (BMI), skeletal muscle mass (SMM), body fat mass (BFM), fat-free mass (FFM), body fat percentage (BFP), body water mass (BWM), waist-hip ratio (WHR), Hospital Anxiety and Depression Scale Anxiety (HADSA), Hospital Anxiety and Depression Scale Depression (HADSD), Beck’s Depression Inventory (BDI)-II, Visual analogue scale (VAS), Fatigue Severity Scale (FSS), Epworth Sleepiness Scale (ESS). Student’s *T*-test. ^§^Fischer’s Exact test. Data has been presented in previous publication (5).

**Table 2:** Baseline data of fasting and non-fasting group (biological mediators).

	FG (*N* = 25)	NFG (*N* = 25)	*p*
	[Median (IQR)]	[Median (IQR)]	
ß-NGF	2.1 (1.7–2.2)	2.1 (1.7–2.4)	0.992
BDNF	591.4 (291.2–860.5)	702.9 (369.7–1001.5)	0.410
GDNF	2.6 (1.8–3.5)	2.6 (1.8–3.3)	0.671
IGF-1	2548.6 (847.2–5583.4)	1760.8 (437.3–3651.3)	0.264
TNF-α	4.5 (3.4–5.4)	4.5 (3.6–5.4)	0.731
IL-8	3.0 (2.0–4.4)	3.7 (2.7–4.5)	0.307
MMP-9	4654.8 (3710.0–5827.2)	3544.0 (2652.1–6347.8)	0.123

All data are presented in median + interquartile range (IQR). ß-nerve growth factor (ß-NGF), brain-derived neurotrophic factor (BDNF), glial cell-derived neurotrophic factor (GDNF), tumor necrosis factor alpha (TNF-α), interleukin-8 (IL-8) and matrix metalloproteinase-9 (MMP-9), IQR: Interquartile range. Student’s *T*-test.

### Effect of RIF on neurotrophic factors

**Figure 2:**
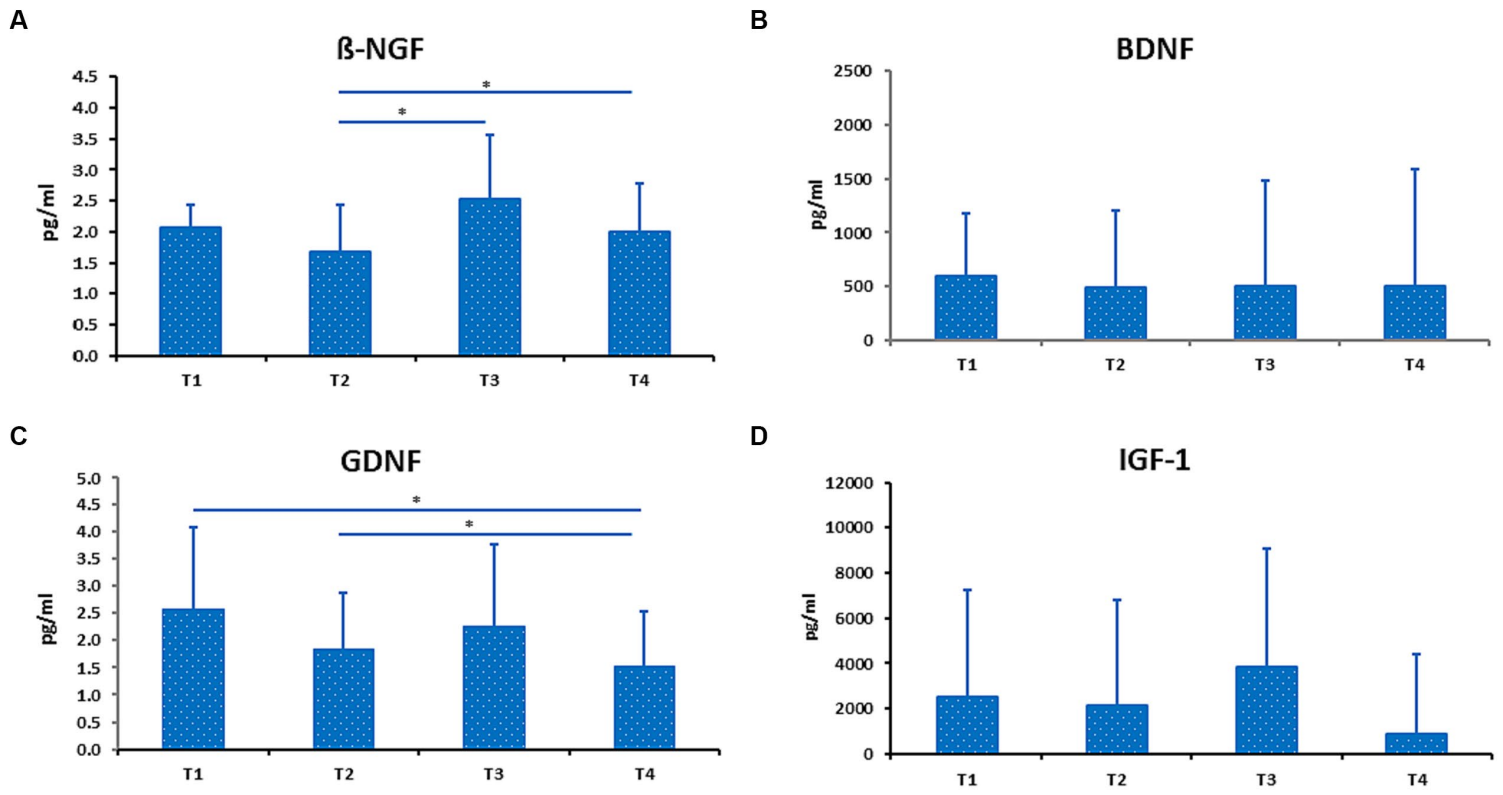
Level of ß-NGF **(A)**, BDNF **(B)**, GDNF **(C)**, and IGF-1 **(D)** in fasting group (FG). Data presented as median + interquartile range (IQR). * *p* < 0.05.

**Figure 3:**
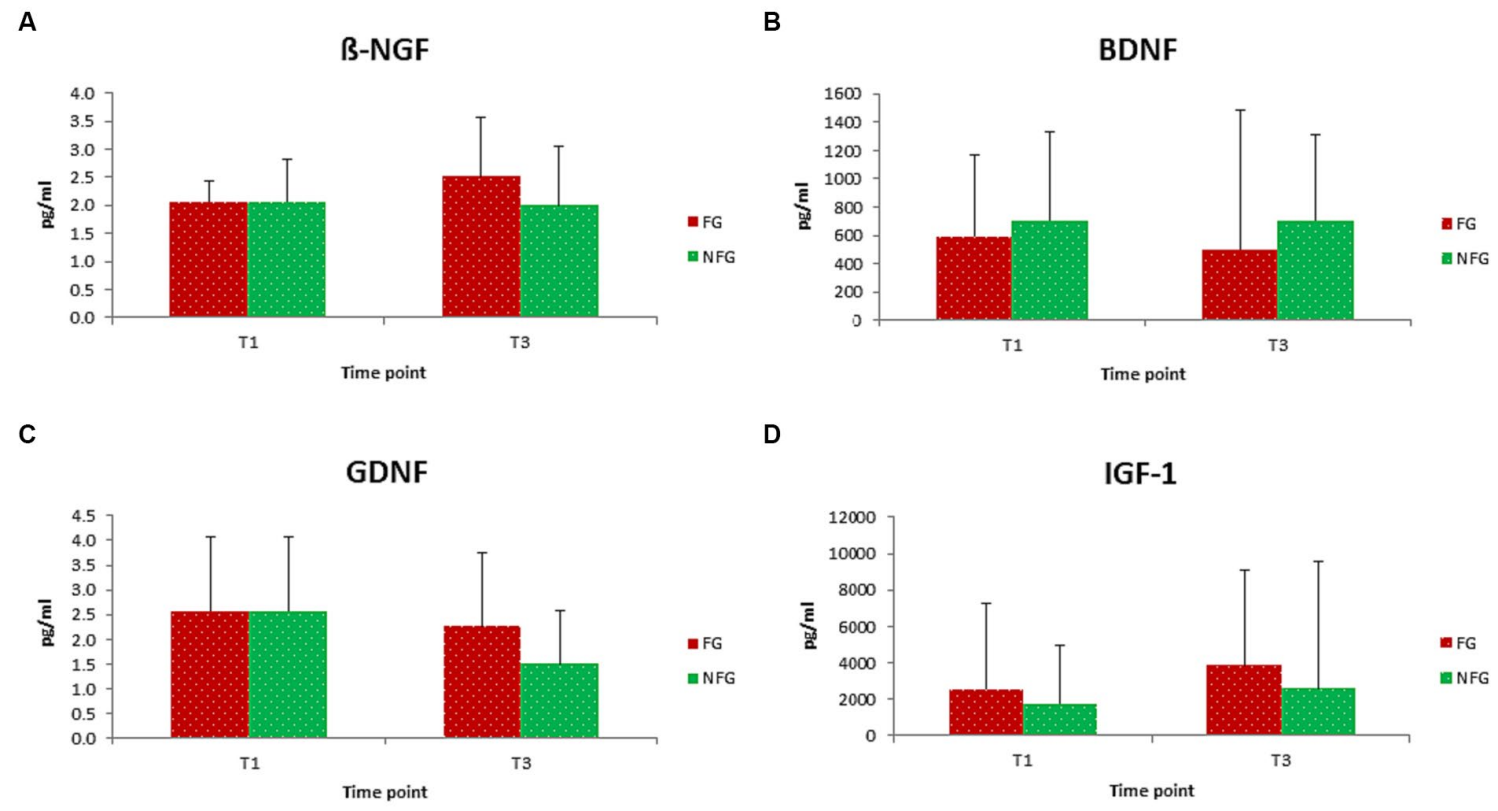
Level of ß-NGF **(A)**, BDNF **(B)**, GDNF **(C)**, and IGF-1 **(D)** in fasting (FG) and non-fasting group (NFG) at T1 and T3. Data presented as median + interquartile range (IQR).

In this study, both ß-NGF and GDNF showed significant changes during RIF in FG only (Figures 2A,C). ß-NGF levels showed significant increased from the middle of Ramadan (T2) to the last days of Ramadan (T3) and one week after the RIF finished (T4). Whereas GDNF levels showed significant decreased from one week before the beginning of Ramadan/baseline (T1) to T4, but also significant decreased from T2 to T4. Furthermore, BDNF and IGF-1 levels showed no significant changes in FG during the study period (Figures 2B,D). In addition, we observed no statistically significant differences between FG and NFG in ß-NGF, BDNF, GDNF and IGF-1 levels from 1 week before RIF (T1) to the last week of RIF (T3) (Figures 3A–D).

### Effect of RIF on IL-8, TNF-a, and MMP-9

**Figure 4:**
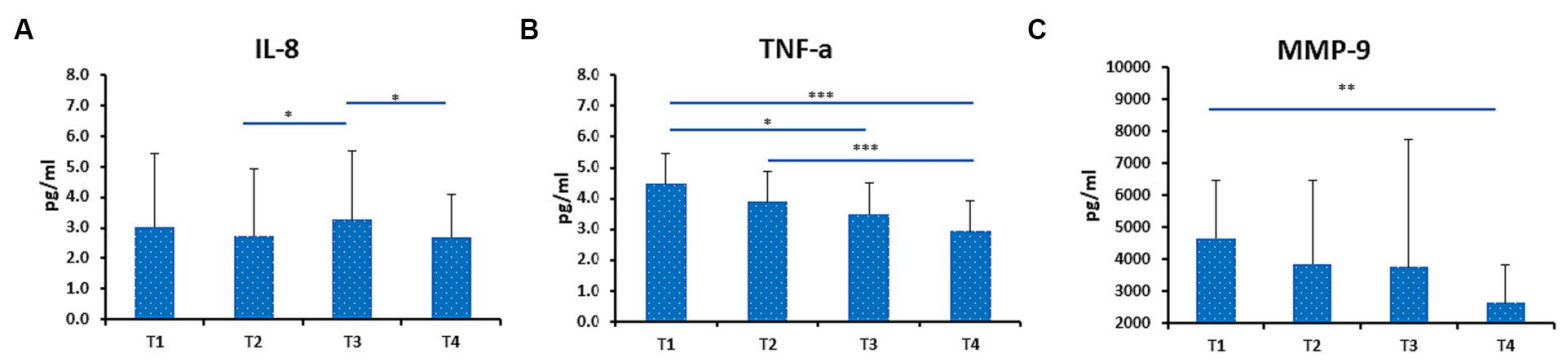
Level of IL-8 **(A)**, TNF-a **(B)** and MMP-9 **(C)** in fasting group (FG). Data presented as median + interquartile range (IQR). Data presented as median + interquartile range (IQR). * *p* < 0.05; ** *p* < 0.01; ***p < 0.001.

In this study, we measured TNF-α and IL-8 levels during IF in healthy male participants in order to determine the effect of RIF on cytokines and chemokines levels. TNF-α levels were significantly decreased from T1 to T3 and T4. TNF-α levels were also decreased from T2 to T4 (Figure 4A). We also observed decreased levels of IL-8 from T3 to T4, although IL-8 levels were increased from T2 to T3 (Figure 4B).

**Figure 5:**
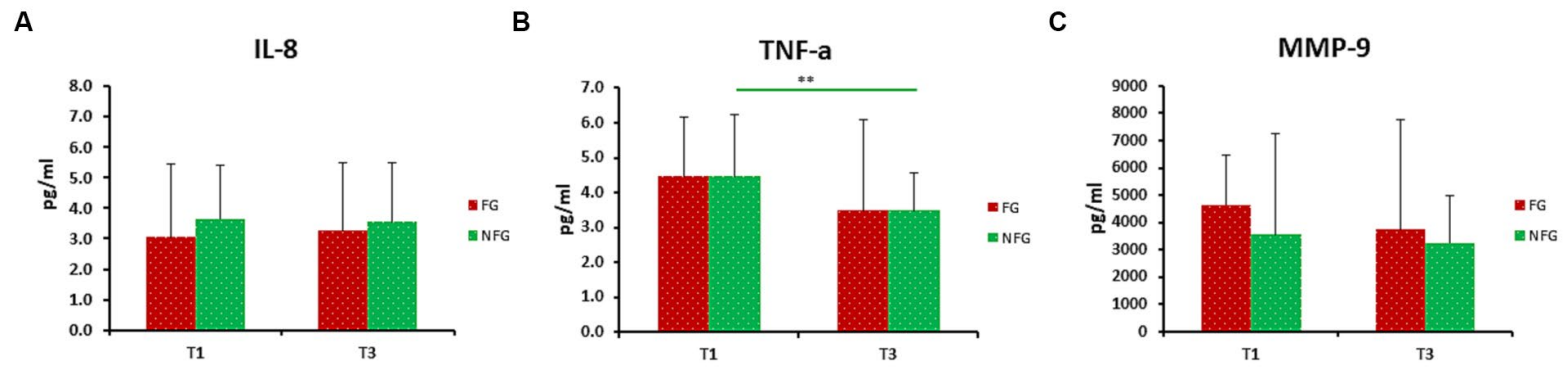
Level of IL-8 **(A)**, TNF-α **(B)**, and MMP-9 **(C)** in fasting (FG) and non-fasting group (NFG) at T1 and T3. Data presented as median + interquartile range (IQR). ** *p* < 0.01.

Furthermore, we measured MMP-9 levels that play an important role in obesity-mediated adipose tissue remodeling. Interestingly, we found that MMP-9 was significant decreased from T1 to T4 in FG (Figure 4C). In addition, we compared the levels of IL-8, TNF-α, and MMP-9 levels of both FG and NFG. No statistically significant differences in IL-8, TNF-α, and MMP-9 were observed at T1 and T3 between FG and NFG (Figures 5A–C). Surprisingly, the TNF-α levels were significantly decreased at T1 and T3 of NFG (Figure 5B).

### Correlations between biological mediators and clinical parameters during RIF

It is of interest to analyze the correlation between clinical parameters and biological mediators (Table 3). In the last days of Ramadan (T3), MMP-9 levels showed a negative correlation with body mass index (BMI). BDNF levels showed negative correlation with Epworth Sleepiness Scale (ESS). Interestingly, TNF-α levels showed positive correlation with Hospital Anxiety and Depression Scale-Anxiety (HADSA) and Hospital Anxiety and Depression Scale–Depression (HADSD). Meanwhile, GDNF and MMP-9 levels showed positive correlation with HADSD.

**Table 3:** Correlation between clinical parameters and biological mediators during RIF in fasting group (*N* = 25).

Time point	Clinical parameter data Mean ± SEM		Biological mediator data Mean ± SEM		*R* and *p*
T3	BMI	24.22 ± 0.73	MMP-9	4477.17 ± 552.28	*R*: −0.446; *p*: 0.025
	ESS	6.68 ± 0.88	BDNF	757.38 ± 126.89	*R*: −0.414; *p*: 0.040
	HADSA	4.16 ± 0.73	TNF-α	3.67 ± 0.31	*R*: 0.398; *p*: 0.049
	HADSD	4.16 ± 0.5	TNF-α	3.67 ± 0.31	*R*: 0.434; *p*: 0.030
			GDNF	2.21 ± 0.22	*R*: 0.526; *p*: 0.007
			MM*p*-9	4477.17 ± 552.28	*R*: 0.419; *p*: 0.037
T4	BW	76.54 ± 2.51	TNF-α	2.18 ± 0.13	*R*: 0.408; *p*: 0.043
	BFM	15.59 ± 1.38	TNF-α	2.18 ± 0.13	*R*: 0.621; *p*: 0.001
			GDNF	1.73 ± 0.13	*R*: 0.668; *p*: 0.000
	WHR	0.89 ± 0.07	GDNF	1.73 ± 0.13	*R*: 0.490; *p*: 0.013
	BDI-II	4.92 ± 1.03	ß-NGF	2.18 ± 0.13	*R*: −0.399; *p*: 0.048

All data are presented in Mean ± SEM. This table shows only the significant correlation data between clinical parameters and biological mediators. Data of clinical parameters of this table has been presented in previous publication (5).

We observed positive correlations of TNF-α to body weight (BW) and body fat mass (BFM) at one week after the RIF finished (T4). GDNF levels had positive correlation with BFM and waist-hip-Ratio (WHR). Interestingly, Beck’s depression inventory (BDI)-II, as depression parameter, showed negative correlation with ß-NGF levels.

## Discussion

The most understanding by common people, the effect of IF is related to the alteration of anthropometric parameters, which include BMI, body weight, and fat loss (5, 30–32). Interestingly, our previous studies, RIF has a positive influence to mental health, fatigue and QoL (5, 30). This effect might be mediated by different biological mediators, particularly cortisol and BDNF (33). Another study also observed that RIF improved body composition and did not exacerbate depression in MDD patients (34). In this current study, we wanted to elucidate the effects of RIF at different time points on neurotrophic factors such as ß-NGF, GDNF, BDNF and IGF-1. Additionally, we also measured other biological mediators such as TNF-α, IL-8 and MMP-9.

In our current study, ß-NGF showed significant increase from the middle of Ramadan (T2) to the last days of Ramadan (T3) and one week after the RIF finished (T4). A comparable study measuring NGF during RIF in woman only participants also showed significant increase of NGF during mid and end of RIF (35). It is known that NGF has an important role in the regulation of neurotransmitters and neuropeptides synthesis of nerve cells (10), but there is no previous evidence on the effect of fasting on NGF levels and it effects to improve brain health through the induction of the NGF levels. Interestingly, Beck’s depression inventory (BDI)-II showed negative correlation with ß-NGF levels. A meta-analysis and systematic review observed that NGF levels were significantly lower in MDD than in healthy control (36).

Furthermore, we observed significantly decreased levels of GDNF from one week before the beginning of Ramadan/baseline (T1) to T4, and significantly decreased levels from T2 to T4. Interestingly, GDNF levels has a positive correlation with HADSD at T3 and BFM and WHR at T4. GDNF levels were shown to be significantly decreased in depression (37) involved in the dopamine system and has been linked to a potential treatment in drug abuse (38). Concerning special psychiatric conditions and its correlation to metabolic levels, a study by Skibinska et al. (39) showed that in psychiatric disorders, such as depression, GDNF and metabolic features such as cholesterol, showed significant correlation between GDNF and cholesterol GDNF. Transgenic mice showed higher brown fat mass, energy expenditure and even increased expression in skeletal muscle. Nevertheless, GDNF does not appear as a predictor for treatment remission in other psychiatric disorders, such as general anxiety disorder (40). However, the findings towards GDNF and its (patho) physiological role remain controversial and further studies need to elucidate the role of GDNF. Best to our knowledge, this study is the first study to analyze the effect of fasting on GDNF.

RIF associated with the decrease of BDNF levels in schizophrenia patients (41). Furthermore, an increased BDNF level was linked to type II diabetes mellitus (T2DM) and obesity (42). A study using rats suggested that IF could potentially protect from T2DM by increasing the levels of BDNF and neurotrophin (NT)3 (43). Interestingly, contradictory results were obtained regarding BDNF levels during RIF in healthy participants. Some studies showed a significant increase of BDNF levels during RIF (35). Whereas another study showed a decrease of BDNF levels during RIF (44). These conflicting reports regarding how IF influence BDNF levels might be because of the different time periods of RIF due to geographical differences and seasonal variations. Different time point of collecting the samples also might resulting the differences. Meanwhile, our previous study showed significantly decreased (33), it could be that the latter study include both male and female participants.

Concerning to insignificant changes of IGF-1-levels during RIF, our results were in line with another study conducted on well trained men during RIF (45), although we cannot compare directly with subjects of this study. In contrast to these findings another study showed significant reduction of IGF-1 levels during RIF by adaptation mechanisms. As shown in mice model, it was hypothesized that short time starvation leads to survival mode and reduction of the growth hormone IGF-1 (46). Reduced IGF-1 levels were linked to extended life span in mice (47).

### Limitation

This study only evaluated the effect of RIF on healthy young males. Therefore, the results could be different in different sex, range of ages (younger/older) and health conditions. We did not include female participants, because they might not fast for the whole of Ramadan due to menstruation. During menstruation periods, females are not obligated to do fasting. It could alter the results. However, we included female participants in other fasting study, although we did not measure similar markers (33). Many biological parameters are quite sensitive to dietary consumption (48–51). Controlling food intake in all participants could lead to better understanding physiological mechanism. However, it could not be done as participants had different cultures, habits and preferences. Otherwise, recording food intake during the study should be done in the future. Assessment of NFG were performed only at T1 and T3, as we hypothesized that NFG would not show any changes during T2 and T4. Main focus of this study, among others, is the difference of FG between before fasting (T1) and the last days of the fasting period (T3). However, we agree that it should be done at all time points for future study.

In summary, RIF as a model of IF could give benefit to health in particular related to mental health and anthropometric parameters. Its benefit is mediated by the alteration of biological parameters, especially neurotrophic factors and cytokines.

## Data availability statement

The raw data supporting the conclusions of this article will be made available by the authors, without undue reservation.

## Ethics statement

The studies involving humans were approved by Ethic Committee of Hannover Medical School (ethics no. 6899; registration code of trial: DRKS00008181). The studies were conducted in accordance with the local legislation and institutional requirements. The participants provided their written informed consent to participate in this study. Written informed consent was obtained from the individual(s) for the publication of any potentially identifiable images or data included in this article.

## Author contributions

SG: Data curation, Investigation, Project administration, Writing – original draft, Writing – review & editing. AS: Data curation, Investigation, Methodology, Software, Validation, Writing – original draft, Writing – review & editing. MB: Data curation, Investigation, Methodology, Software, Writing – review & editing. IH: Data curation, Investigation, Project administration, Writing – review & editing. GG: Funding acquisition, Resources, Writing – review & editing. CG: Funding acquisition, Resources, Writing – review & editing. BN: Conceptualization, Data curation, Formal analysis, Investigation, Methodology, Software, Supervision, Validation, Visualization, Writing – original draft, Writing – review & editing.
